# Deletion of Parasite Immune Modulatory Sequences Combined with Immune Activating Signals Enhances Vaccine Mediated Protection against Filarial Nematodes

**DOI:** 10.1371/journal.pntd.0001968

**Published:** 2012-12-27

**Authors:** Simon A. Babayan, HongLin Luo, Nick Gray, David W. Taylor, Judith E. Allen

**Affiliations:** 1 Centre for Immunity, Infection and Evolution, University of Edinburgh, Edinburgh, United Kingdom; 2 Institute of Immunology and Infection Research, University of Edinburgh, Edinburgh, United Kingdom; 3 Division of Pathway Medicine, School for Biomedical Studies, University of Edinburgh, Edinburgh, United Kingdom; Uniformed Services University, United States of America

## Abstract

**Background:**

Filarial nematodes are tissue-dwelling parasites that can be killed by Th2-driven immune effectors, but that have evolved to withstand immune attack and establish chronic infections by suppressing host immunity. As a consequence, the efficacy of a vaccine against filariasis may depend on its capacity to counter parasite-driven immunomodulation.

**Methodology and Principal Findings:**

We immunised mice with DNA plasmids expressing functionally-inactivated forms of two immunomodulatory molecules expressed by the filarial parasite *Litomosoides sigmodontis*: the abundant larval transcript-1 (LsALT) and cysteine protease inhibitor-2 (LsCPI). The mutant proteins enhanced antibody and cytokine responses to live parasite challenge, and led to more leukocyte recruitment to the site of infection than their native forms. The immune response was further enhanced when the antigens were targeted to dendritic cells using a single chain Fv-αDEC205 antibody and co-administered with plasmids that enhance T helper 2 immunity (IL-4) and antigen-presenting cell recruitment (Flt3L, MIP-1α). Mice immunised simultaneously against the mutated forms of LsALT and LsCPI eliminated adult parasites faster and consistently reduced peripheral microfilaraemia. A multifactorial analysis of the immune response revealed that protection was strongly correlated with the production of parasite-specific IgG1 and with the numbers of leukocytes present at the site of infection.

**Conclusions:**

We have developed a successful strategy for DNA vaccination against a nematode infection that specifically targets parasite-driven immunosuppression while simultaneously enhancing Th2 immune responses and parasite antigen presentation by dendritic cells.

## Author Summary

Filarial infections are endemic in more that 80 countries, affecting over 120 million people and putting 1 billion more at risk. Antifilarial drugs must be administered regularly to infected people to control the disease, but they are contraindicated in under 6 year-olds and in pregnant women. Further, reports of drug resistance are now accumulating. A vaccine would therefore greatly help fight these diseases. Live attenuated L3 filariae larvae can evoke a protective immunity but their production is impractical and use in humans unacceptable while the efficacy of sub-unit vaccines has been poor. Filariae secrete proteins capable of suppressing their host's immune response, and have the potential to interfere with immunisation. We therefore decided to vaccinate hosts against secreted parasite products that modulate host immune responses rather than against structural components of the worms, and to boost the host's immune system by directly enhancing the uptake of parasite material by antigen presenting cells. This strategy generated substantial protection against both adult and offspring of a filarial parasite in mice. This provides a strong proof of principle for the anti-immunomodulatory approach we have developed.

## Introduction

DNA vaccination is a promising technology that is being developed to combat diseases such as flu, HIV, and cancer [1]. Furthermore, the stability and relatively low production cost of DNA vaccines provide hope for treating individuals in developing countries. After more than 20 years of mass drug treatment programs based on a limited choice of drugs, lymphatic filariasis and onchocerciasis remain major public health problems. Control programs in some areas are now threatened by the emergence of drug-resistance [2], [3], while in communities where both onchocerciasis and loiasis are endemic, mass treatment with ivermectin is contraindicated because of the risk of severe side effects associated with death of *Loa loa* microfilariae [4]. These circumstances argue strongly for the development of vaccines to complement drug treatment strategies.

Filarial nematodes are tissue-dwelling parasites that can be killed by exposure to Th2-mediated effector mechanisms, with eosinophils and antibody particularly critical for protection against re-infection [5]. However, these parasites establish chronic infections in a large number of species, including ∼150 million humans in whom immunopathological reactions cause a spectrum of clinical diseases. The persistence of filarial parasites has been shown to be enabled, in part, by excretory/secretory (ES) products [6]–[10]. As a consequence, the efficacy of a vaccine against filarial infections is likely to depend on how well it disrupts parasite immune evasion mechanisms. Given that the maintenance and transmission of filarial infections requires very few adult parasites [11] and that our previous work suggests that they are able to increase their fecundity in response to host immune attack [12], any intervention strategy should be assessed by its ability to suppress the transmissible stages, the microfilariae. Not only is this critical to reducing disease transmission, but in onchocerciasis microfilariae are the main cause of pathology.

The rodent filarial nematode, *Litomosoides sigmodontis*, has been used to elucidate many of the regulatory pathways that allow parasite establishment and is ideally suited for assessing vaccination success against filarial nematodes as the parasites can develop patent infections in BALB/c mice [5], [13]. The infective larvae are inoculated subcutaneously, and over 4–6 days migrate to the pleural cavity, where they mature to adulthood, mate and produce microfilariae that enter the bloodstream by 60 days post-infection. Consequently, protective immunity against all stages of the parasite's lifecycle can be assessed. We tested the vaccine potential of two *L. sigmodontis* immunomodulatory ES products by expression of these proteins in DNA vaccine constructs designed to improve antigen presentation and enhance Th2 immune responses [14], [15].

While there have been previous attempts to immunise rodents against filarial nematodes using DNA vaccines [16]–[18], this is the first study to do so that specifically targets parasite-driven immune modulation and uses a filarial parasite that produces patent infections in laboratory mice, thus allowing manipulation and deeper examination of host immunity through methodology developed in inbred mice.

## Materials and Methods

### Ethics statement

All procedures involving animals were approved by the University of Edinburgh ethical review committee, and performed under license from the UK Home Office in accordance with the Animals (Scientific Procedures) Act 1986.

### Mice and parasites

All immunisations and infections were performed with female BALB/c mice, starting at ages of 6–7 weeks. Mice were housed in individually ventilated cages and infected subcutaneously with 30 or 40 *L. sigmodontis* infective larvae (iL3). Two experiment endpoints were chosen based on the life cycle of *L. sigmodontis*, D10 post-inoculation (p. i. ) when most larvae will have reached the L4 stage; and, at D60 p. i. after the onset of the patent phase.

### Cloning and mutation of LsALT and LsCPI

All cloning was carried out following the recommendations of the pcDNA 3.1 Directional TOPO Expression Kit (Invitrogen). LsALT (DQ451171.1) and LsCPI (AF229173.1) were amplified from a cDNA preparation of *L. sigmodontis* iL3 with the primers detailed in Table S2. The desired mutation of asn66 to lys66 in LsCPI was generated with the QuikChange Site Directed Mutagenesis Kit (Stratagene) using primers detailed in Table S2. Fusion constructs containing single-chain anti-DEC205 antibody (DEC) upstream of our target antigen were produced from ready-made constructs kindly provided by Dr. Ralph Steinman [19]. Briefly, PCR products of genes of interest were digested with NotI and XbaI (Neb laboratory, UK), then ligated into an NotI and XbaI-digested anti-mouse dec-205 single chain antibody - ovalbumin construct (DEC-OVA) or antibody control Ig-OVA to replace the fragment of OVA gene, respectively. All plasmids were sequenced to confirm identity.

### Protein expression for ELISA

LsALT and LsCPI were cloned into pET21b and pET29c respectively. Both plasmids were made with PCR products derived from primers spanning the full CDS minus the stop codon and with flanking restriction enzyme recognition sites. The recombinant CPI and ALT containing a T7 tag at the N terminus and His tag at the C terminus were produced in the *E. coli* BL21(DE3) strain (Novagen UK) and purified on His- binding resin columns using automated AKTAprime (Amersham Pharmacia).

### Immunisations and challenge

Plasmids were injected in the tibialis anterior muscle of the left leg with a 27G needle, immediately followed by electroporation with an ECM 830 generator+Tweezertrodes (BTX Harvard Apparatus) using as settings 8 pulses, 200 V/cm, 40 ms duration, 460 ms interval [20]. Each mouse was immunised twice separated by 2 weeks interval with 40 µg of DNA total made up by equal quantities of each plasmid species, delivered in 50 µl PBS. As a consequence, the quantity of each individual plasmid was reduced as the number of different plasmids incorporated into the inoculums increased. However, the quantity of each one remained in excess of the minimal efficient dose [21]. *In vivo* expression of the gene of interest was determined by the detection of mRNA with specific primers 4 weeks after immunisation (Figure S1 and Table S3). Mice were challenged 4–6 weeks after the last immunisation.

### Immunological read-outs

Blood was collected from individual mice after the first immunisation, then every other week to measure the increase in antibody titres. At experiment end point cells were recovered from thoracic lymph nodes for antigen recall assays of specific cytokine production and proliferation. Pleural lavage fluid was also collected for cytokine and cellular infiltrate measurement. Cytokines IL-4, IL-5, IL-10, IFN-γ and IL-13, and total IgE levels were measured by sandwich ELISA, and specific anti-*L. sigmodontis* IgG1 and IgG2a responses were measured by indirect ELISA against whole soluble extract coated at 10 µg/ml, and anti-LsALT or anti-LsCPI antibodies against the respective native recombinant proteins coated at 5 µg/ml using the antibody pairs described elsewhere [22] and detected with TMB-H_2_O_2_+(KPL) at 450 nm. Titres were assessed by two fold serial dilution of the serum, and determined as the highest dilution factor for which O. D. values exceeded 3 standard deviations above control wells from the same plate. All ELISAs were repeated at least once. The 1/800 O. D. was used in some analyses.

### Parasitological read-outs

Parasite survival was determined at experiment endpoint. Adult filariae were isolated from the pleural cavity lavage fluid in10 ml cold PBS, fixed in hot 70% ethanol and counted. Protection was calculated as (mean burden in primary infected animals - mean burden of vaccinated animals)/mean burden in primary infected animals. Microfilariae were counted in 30 µl of blood after fixation in 570 µl of BD FACS lysing solution (BD Biosciences) under an inverted microscope.

### Data analysis

Generalised linear models (GLM) were used to compare the effects of different vaccine formulations on parasitological and immune parameters as they allow more flexibility in specifying the distribution of response variables and better model fitting through Maximum Likelihood estimation. Parasite and cell counts were modelled assuming poisson or negative binomial error distributions; antibody levels were log-transformed. Homoscedasticity and normality of the residuals were assessed with the Fligner-Killeen test of homogeneity of variances and the Shapiro-Wilk normality test, respectively. The non-parametric Kruskal-Wallis rank sum test followed by pairwise comparison with the Wilcoxon rank sum test with Bonferroni correction for multiple comparisons were applied to data that didn't conform to a standard distribution. Average parasite counts per group were used to calculate pairwise measures of protection. A principal component analysis (PCA) was performed to identify major trends across the large number of immunological measures performed. Briefly, data were scaled to null mean and unit standard deviation, and the broken-stick criterion was then used to select the principle components having the best explanatory power [23]. The biological interpretation of the resulting PC was then based on the relative contribution of each immune factor to it, e.g. a preponderance of Th2-associated factors or of pro-inflammatory factors. Individuals' predicted values from the PCA were then used as explanatory variables in a GLM to assess which components were best correlated with parasite killing. Statistical analyses are reported in figures and legends only when significant effects of vaccine formulations were found. All analyses and graphs were performed in R 2.15 [24].

## Results

### Suppression of ALT-driven immune modulation enhances immunisation

LsALT is the most abundant transcript produced by the infective larvae of filarial nematodes [25], [26], and is suspected of modulating the host Th2 immune responses [9]. ALT's potential as a vaccine has been tested by several laboratories using different filarial and host species and has shown variable success in inducing immunity [17], [25], [27]–[31]. As a starting point for this study the immunisation efficacy of plasmids containing *L. sigmodontis* antigens was enhanced by electroporation into skeletal muscle [32]. However, our attempts using plasmids expressing LsALT (ALT) to immunise mice against *L. sigmodontis* failed to induce Th2 responses. We thus wanted to improve immunisation efficacy against LsALT by specifically enhancing Th2 responses with IL-4, and by enhancing dendritic cell recruitment and activation. Levels of IgG2a, IgG1and IgE were measured to determine the relative balance between Th1 and Th2 responses while IL5 production and eosinophil numbers where used as measures of vaccine-induced immunity against the filariae [33], [34].

**Figure 1:**
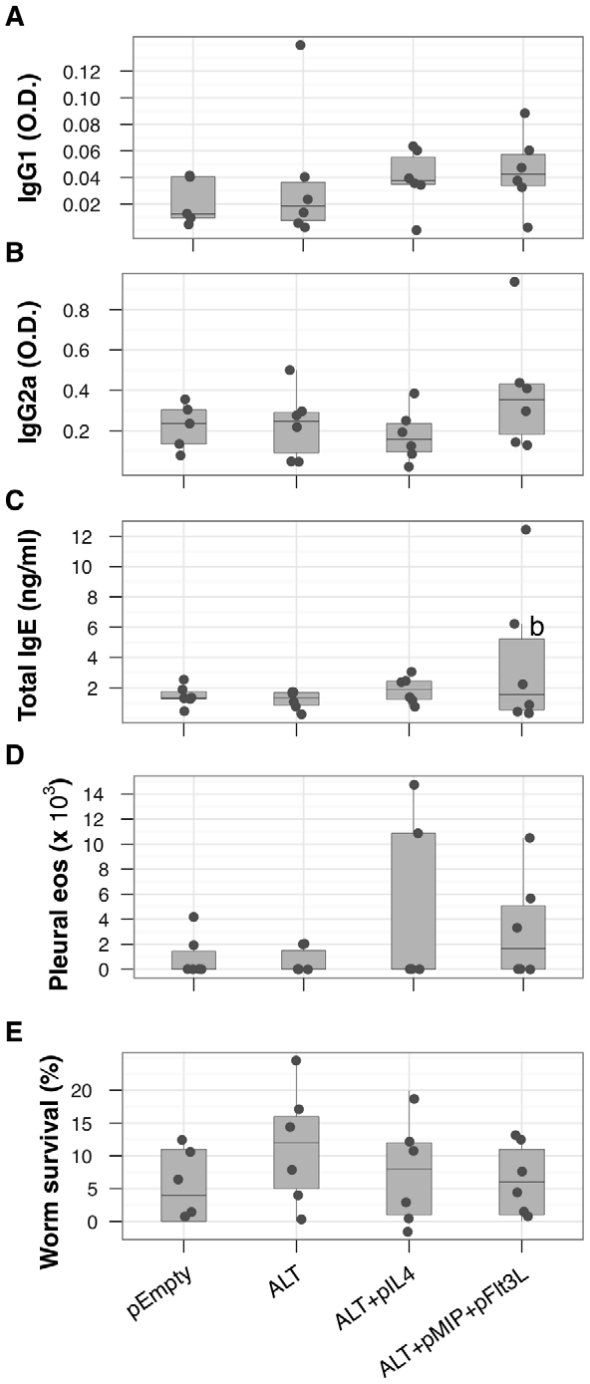
Coinjection of plasmids encoding IL-4 and MIP-1α+FLT3L marginally improves immunisation with LsALT-expressing plasmids. Mice were immunised with DNA plasmids expressing parasite LsALT (ALT) and murine IL-4 (pIL4) or MIP-1α (pMIP) and Flt3L (pFlt3L). Empty expression plasmid (pEmpty) was injected as a control for DNA-induced inflammation to equalise DNA quantities between groups. All groups were subsequently challenged with live infective *L. sigmodontis* larvae subcutaneously and infection was allowed to progress for 10 days. (A–B), LsALT-specific IgG1 and IgG2a, respectively and (C), concentrations of total IgE in the serum were increased by the coadministration of pMIP and pFlt3L compared to ALT alone (b, *P* = 0.03). (D), Eosinophil recruitment to the site of infection, the pleural cavity. Cell enumerations were performed on pleural lavage fluid. (E), Parasite survival represented as the proportion of worms found in the pleural cavity of infected mice relative to the infective dose. Points represent individual mice at D10 p.i. (N = 5–6 mice per group).

BALB/c mice immunised with ALT and challenged with infective *L. sigmodontis* larvae, failed to generate LsALT-specific IgG responses or local eosinophil recruitment ten days after challenge. Although not reaching significance, the co-administration of plasmids encoding IL-4 (pIL4) increased LsALT-specific IgG1 but not IgG2a levels, while antigen presenting cell activators MIP-1α (pMIP) and Flt3L (pFlt3L) [35] marginally increased both IgG1 and IgG2a (Figure 1A–B).

Coinjecting ALT with pMIP+pFlt3L significantly increased the production of total IgE production compared to ALT alone (Figure 1C). There was an indication that eosinophil recruitment to the site of infection was also increased by the addition of pIL-4 and pMIP+pFlt3L compared to ALT alone (Figure 1D). The vaccine containing ALT alone resulted in non-significantly higher parasite survival than in pEmpty controls (Figure 1E), while the addition of pIL4 or pMIP+pFLt3L seemed to counter this facilitation. Overall, no protection was detected at day 10 post-challenge.

**Figure 2:**
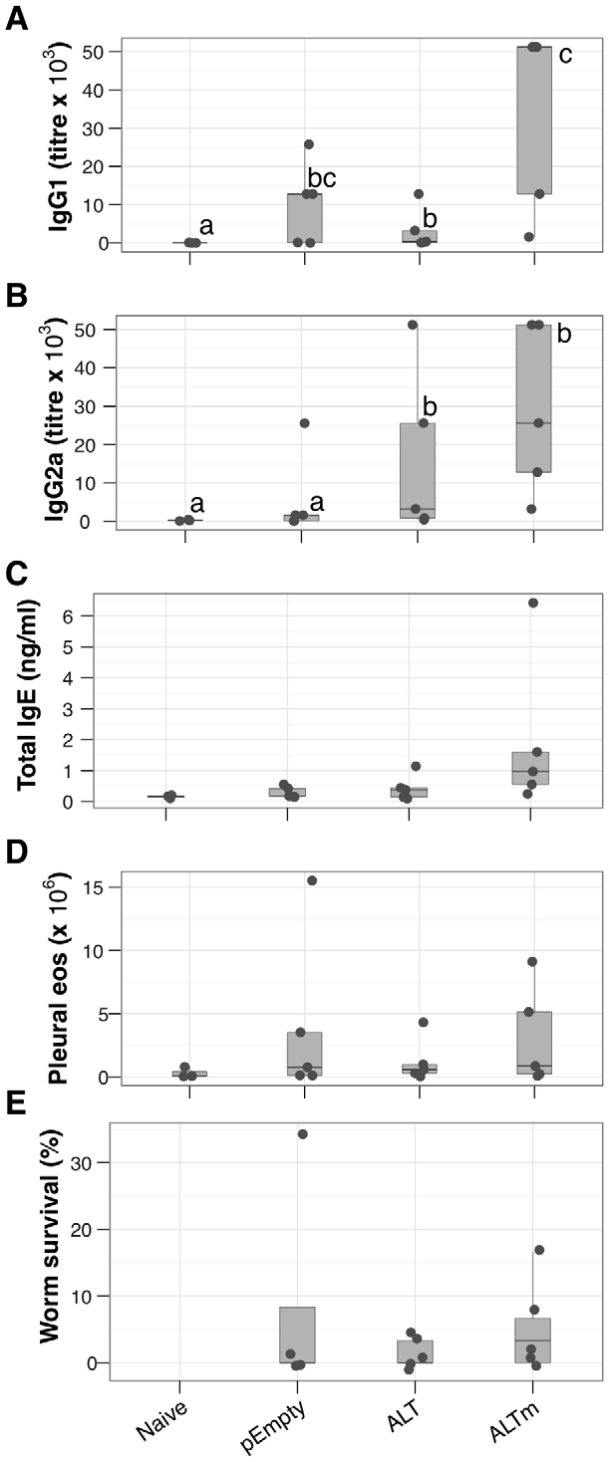
DNA immunisation with inactivated LsALTm enhances Ls-ALT-specific humoral responses. Mice were immunised with DNA plasmids expressing the native sequence of LsALT (ALT), the acidic domain-deleted form (ALTm), or nothing (empty expression plasmid pEmpty). All groups except naïve mice were challenged with live infective *L. sigmodontis* larvae and the infection was allowed to progress for 10 days. (A–B), LsALT-specific IgG1 and IgG2a concentrations in serum (A: c vs. [a, bc, b], *P* = 0.002, 0.1, 0.05; B: a vs. b, *P*≤0.02). (C), Total serum IgE levels. ELISAs were performed with the complete recombinant LsALT. (D), Total eosinophil numbers in the pleural cavity. (E), Parasite survival represented as the proportion of worms found in the pleural cavity of infected mice relative to the infective dose (except naive mice). Points represent individual mice (N = 5 mice per group).

These data showed that ALT could generate specific antibodies, but that it was poorly immunogenic, and possibly immunomodulatory. Previous data have suggested that the variable acidic domain of *Brugia malayi* ALT-2 confers the protein its immune modulatory properties [9]. We thus decided to generate a mutated LsALT lacking the acidic domain (ALTm) in the hope that this would enhance LsALT-specific immune responses to the remaining epitopes. Immunising with ALTm resulted in a significant increase in LsALT-specific IgG1 production above naïve controls and a marginal increase above the native form ALT (Figure 2A), consistent with the hypothesis that the deletion of the acidic domain would overcome LsALT-driven immunosuppression. The ALTm vaccine induced a strong increase in LsALT-specific IgG2a concentrations above naïve controls (Figure 2B), but no further increase above the native form ALT. Although total IgE production and eosinophil recruitment were higher in the ALTm group than in the ALT group, this was not statistically significant (Figure 2C–D). Despite relatively high antibody production in several repeats of this experiment, significant protection against a challenge infection was never achieved (Figure 2E).

**Figure 3:**
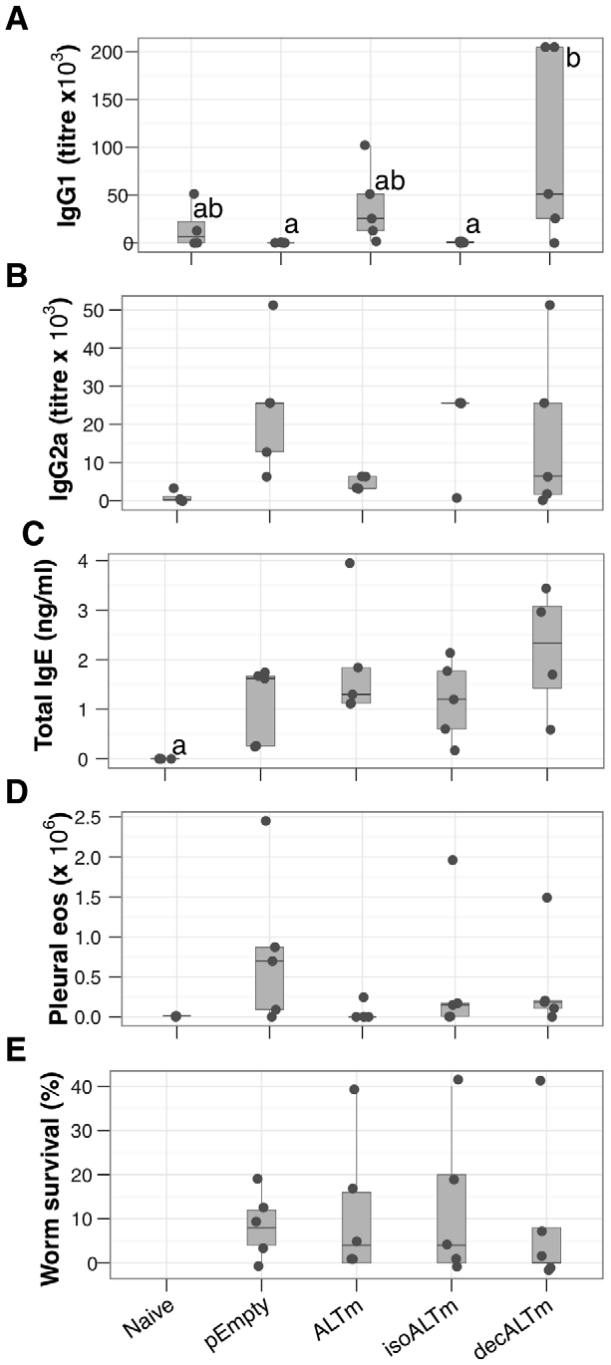
Dendritic-cell targeted ALTm enhances humoral and cellular responses against LsALT. The sequence encoding ALTm was fused to the sequence for a single-chain antibody targeting DEC205 (decALTm) or to a control antibody sequence which was derived from an antibody of the same isotype that does not bind to DCs). All vaccinated mice were also injected with pIL-4, pMIP and pFlt3L. All groups except naïve mice were challenged with live infective *L. sigmodontis* larvae. The data shown are representative of results obtained at D10 and at D60 p.i. (A) Serum LsALT-specific IgG1 levels were significantly increased in the ALTm and decALTm groups relative to the control plasmids (b vs. [a, ab], *P*≤0.05, 0.8). (B) LsALT-specific IgG2a concentrations in serum were highly variable. ELISAs were performed with the wild-type recombinant LsALT. (C) Targeting ALTm to dendritic cells did not increase total total serum IgE or (D), eosinophil recruitment to the site of infection but IgE was significantly higher than in naïve controls (C, *P* = 0.02). Cytokines and cell enumerations were performed on pleural lavage fluid. (E), Parasite survival represented as the proportion of worms found in the pleural cavity of infected mice relative to the infective dose (except naive mice). pEmpty, non-coding plasmid control; ALTm, plasmid encoding the acidic domain-deleted sequence of LsALT; isoALTm, plasmid encoding a non-specific scFv control-ALTm construct; decALTm, plasmid encoding the anti-DEC205 scFv-ALTm construct. Points represent individual mice (N = 5 mice per group).

In an attempt to enhance the protective potential of ALTm, a plasmid was constructed to encode a fusion protein comprising ALTm and an anti-DEC205 single chain Fv antibody (decALTm). The expressed fusion protein directly binds dendritic cells through the DEC205 surface receptor [19]. A second plasmid encoding a fusion protein comprising an antibody with irrelevant specificity and ALTm (isoALTm) was constructed for use as a control. For these experiments we chose to include pIL-4, pMIP and pFlt3L in all vaccine formulations to maximise their protective potential. Immunising with decALTm resulted in increased LsALT-specific IgG1 and IgG2a concentrations in 2 out of 5 mice, but did not increase total IgE or eosinophilia above the non-DC-targeted form, ALTm (Figure 3A–C). Despite demonstrable enhancement of IgG1 antibody levels by both ALTm and decALTm compared to empty vector, no statistically significant reduction in parasite numbers was detected at day 60 post-challenge (Figure 3E). This suggests that immunising against LsALT alone was not sufficient to evoke protective immunity.

### Suppression of CPI-2-driven immune modulation enhances immunisation

Because removal of immune modulatory sequences from LsALT had a significant impact on its ability to induce an immune response, we decided to apply a similar approach to another immune modulatory filarial protein. Filarial cystatins are potent downregulators of inflammation [36] and antigen processing by host cells, as shown for CPI-2 from *B. malayi* which inhibits asparaginyl endopeptidase activity [37]. We isolated the *L. sigmodontis* homologue, LsCPI, and cloned it into the same pcDNA3.1 vector as LsALT, either in its native sequence (CPI) or with a mutation that disrupts its AEP inhibitory activity (CPIm).

**Figure 4:**
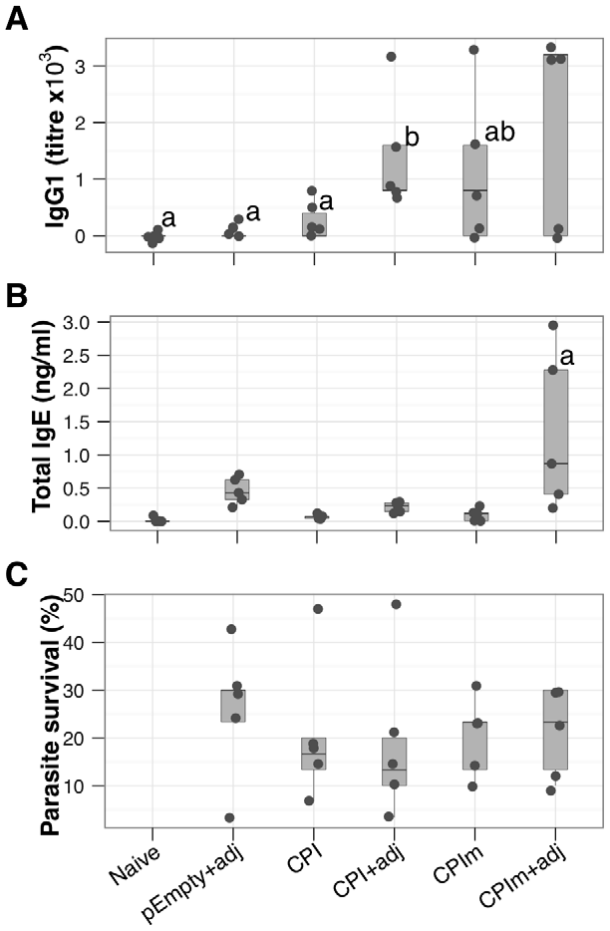
Removal of asparaginyl endopeptidase inhibition by LsCPI increases its immunogenicity. Mice were immunised against LsCPI with plasmids encoding either the native protein (CPI) or a plasmid expressing its mutated form (CPIm), in combination with pIL4+pFlt3L+pMIP or alone. All mice other than naïve were challenged with live infective *L. sigmodontis* larvae. (A), LsCPI-specific IgG1 (b vs. [a, ab], *P* = 0.02, 0.3), and (B), total serum IgE concentrations (a, *P*≤0.001). ELISAs were performed with recombinant LsCPI. (C), Parasite survival 60d after infection. pEmpty, non-coding plasmid control; CPI, pcDNA 3.1 plasmid with an insert encoding LsCPI; CPIm, mutated form of LsCPI; adj = pIL4+pFlt3L+pMIP. Points represent individual mice at D60 p.i. (N = 5 mice per group).

**Figure 5:**
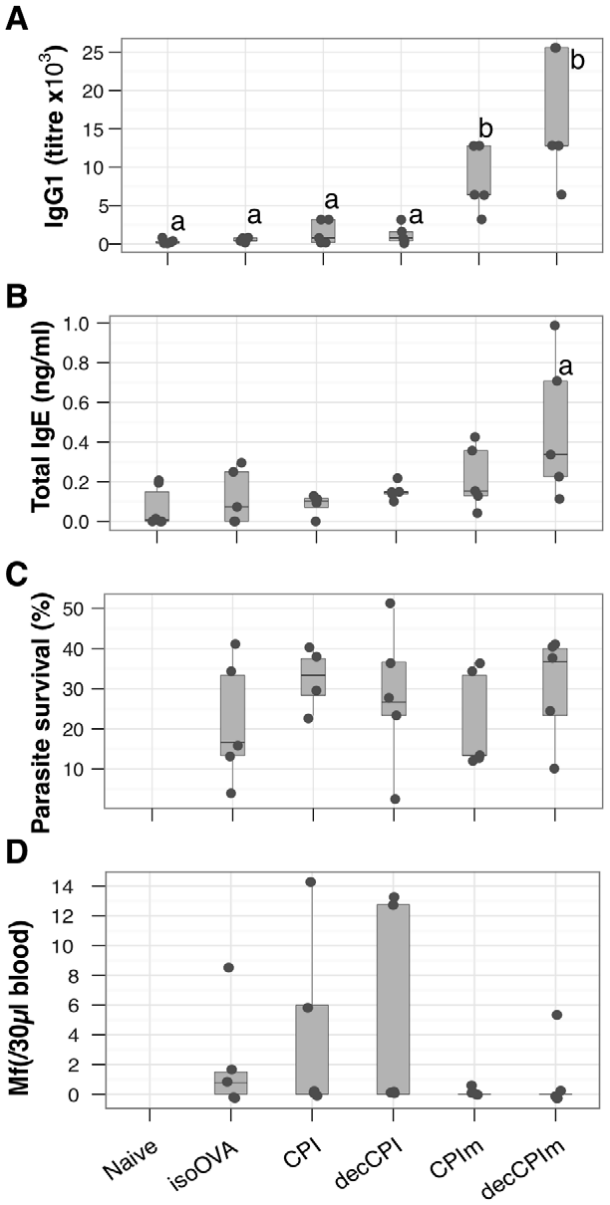
Enhancing type 2 immunity against LsCPI by targeting DCs. Mice were immunised with plasmids containing the CPI, decCPI, CPIm or decCPIm construct. (A), LsCPI-specific IgG1 concentrations (b vs. a, *P*≤3×10^−5^), (B), total IgE serum concentrations (a, *P*≤0.05), and (C–D) adult parasite survival and microfilariae peripheral blood densities. All groups except naïve received pIL4+pFlt3L+pMIP. isoOVA, control plasmid expressing OVA fused to an isotype control for sc-Fv-DEC205; CPI, pcDNA 3.1 plasmid with an insert encoding LsCPI; CPIm, mutated form of LsCPI. Points represent individual mice at D60 p.i. (N = 5 mice per group).

To verify our previous findings with immune enhancing plasmids the native and modified constructs of LsCPI were administered with or without pIL4+pFlt3L+pMIP (indicated as +adj in figure 4). LsCPI-specific IgG1 production was significantly enhanced by the co-administration of pIL4+pFlt3L+pMIP (Figure 4A). The combination of the mutation of CPI and pIL4+pFlt3L+pMIP significantly enhanced the production of IgE compared to all the other groups (Figure 4B). However, no protection from challenge infection was achieved by the vaccine regimens above (Figure 4C). We therefore followed the DC-targeting strategy used with ALT: CPI and CPIm were fused with a scFv-DEC205 sequence (decCPI and decCPIm, or isoCPI for scFv isotype control). All groups received pIL4+pFlt3L+pMIP, as this had also improved immunisation against LsALT (Figure 3). In this experiment, both the mutation of CPI and the targeting of dendritic cells enhanced immune responses to LsCPI. The resulting CPIm and decCPIm constructs induced strong increases in LsCPI-specific IgG1 compared to isoOVA-, CPI- and CPIm-immunised animals (Figure 5A) and in IgG2a compared to CPI (Figure S2A) as well as total IgE (Figure 5B). Nonetheless, there was no significant protection as neither adult worm recoveries (Figure 5C) nor microfilariae densities (Figure 5D) differed statistically between control mice and mice immunised with CPIm or decCPIm. However, the mutation of CPI showed a trend towards reduced microfilariae in the peripheral circulation (Figure 5D).

### Protective immunity achieved by dual modified antigen vaccine

**Figure 6:**
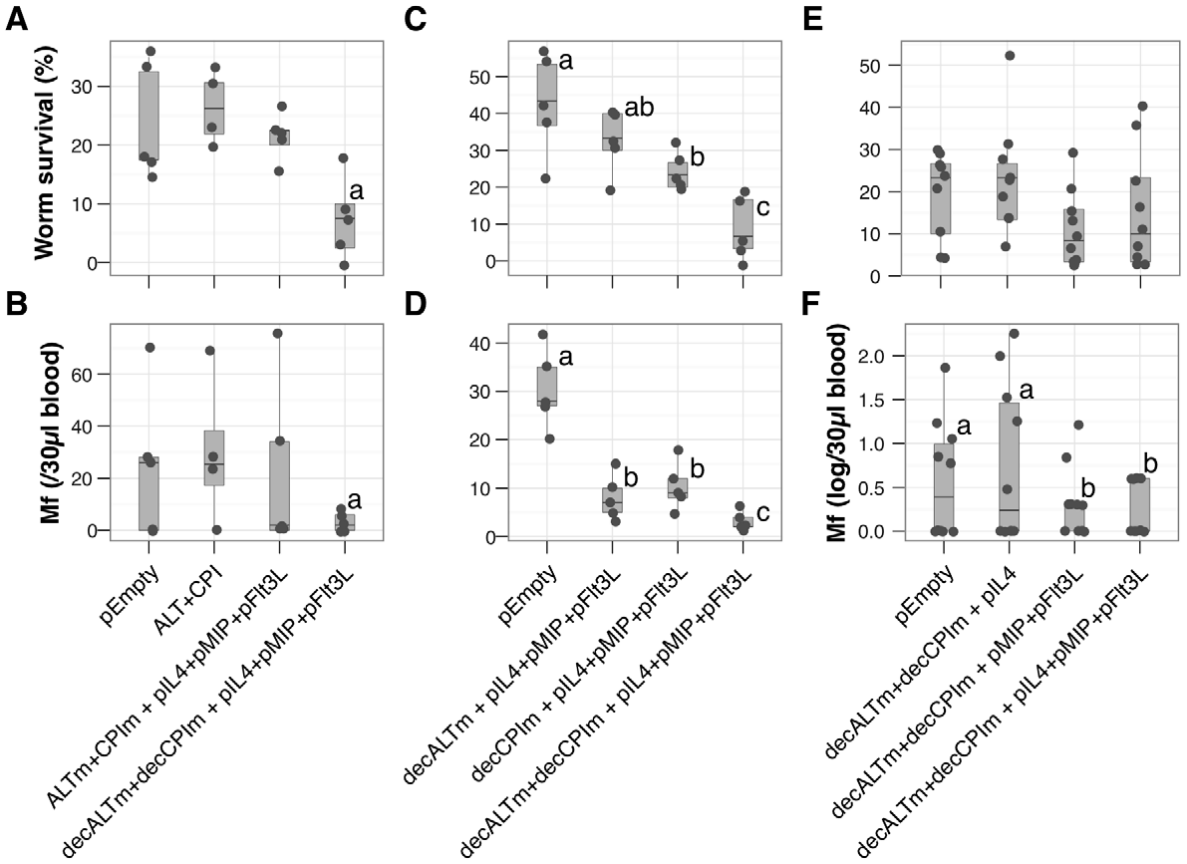
Dual modified antigens in combination with cytokine-expressing plasmids generate protective immunity. Mice were immunised with decALTm, decCPIm, or both in addition to the adjuvant combinations pIL4+pFlt3L+pMIP, pIL4 alone or pFlt3L+pMIP. (A–B), Comparison of dual unmodified antigens and dual modified antigen formulations. (C–D), comparison of single modified vs. dual modified vaccine protective capacity. (E–F), comparison of Th2- and antigen presenting cell-promoting plasmids. (A, C, E), Proportion of adult parasites present in the pleural cavity of mice after 60 days of infection relative to infection dose (A: a, *P*<4×10^−13^; C: c vs. [b, ab, a], *P* = 0.002, 4×10^−5^, 3×10^−7^). (B, D, F), number of microfilariae present in 30 µl of peripheral blood circulation (B: c vs. [b, a], *P* = 0.004, 8×10^−14^; F: b vs. a, *P*_d_<0.04). pEmpty, non-coding plasmid control; decALTm, plasmid encoding the anti-DEC205 scFv-ALTm construct, decCPIm, plasmid encoding the anti-DEC205 scFv-CPIm construct, pIL4 pMIP and pFlt3L, plasmids expressing mouse IL-4, MIP-1α and Flt3L respectively. Points represent individual mice at D60 p.i. (A–D, N = 4–5 mice per group; E–F N = 9–10 mice per group).

On their own neither ALT or CPI induced a substantial protective effect. However, recombinant vaccines can work more effectively in combination [16], [27], [38]–[40] and indeed in a cattle model of onchocerciasis both ALT and CPI were part of a cocktail of recombinant proteins that generated protection against natural challenge [41]. We thus chose to use a combination of both parasite antigens along with the full complement of ‘adjuvant’ plasmids in an effort to increase the level of protection. Significant protection was achieved when mice were immunised with dual modified parasite antigens and the full combination of cytokine-expressing plasmids (Figure 6). Adult parasite numbers were reduced by 71% in the mice that received the full modified vaccine relative to those that received unmodified antigens ALT+CPI, by 65% compared to those that received ALTm+CPIm+adjuvants and by 68% compared to those that received only empty plasmids (Figure 6A). Average microfilaraemia in both the full vaccine and the ALTm+CPIm groups was reduced by over 85% compared to both the ALT+CPI group and empty plasmid controls (Figure 6B). The lack of protection in the ALT+CPI group suggests that combining vaccine targets is not sufficient on its own. Further, the benefit of targeting the antigens to dendritic cells via DEC205 was to induce more rapid killing of adult parasites.

The importance of the ‘adjuvants’ was confirmed in a separate experiment. Significant protection was achieved by D60 post-challenge in the groups that received the dual vaccine decALTm+decCPIm, reaching an average 82% reduction of adult parasite burden relative to non-vaccinated animals (Figure 6C), and a 90% reduction of circulating microfilariae (Figure 6D). The dramatic effect on microfilariae numbers without full removal of the adult population suggested that at least some of the vaccine impact was occurring late in infection and was specifically targeting larval stages or worm fecundity.

To determine the relative contribution of the ‘adjuvant’ plasmids to protection, mice were immunised with decALTm+decCPIm with either pIL4, pMIP+pFlt3L, or both as in the full vaccine. While the reduction in adult worm counts by the full vaccine formulation reached 57%, the formulation with pIL4 alone showed no protective effect relative to pEmpty controls, and the formulation containing MIP+pFlt3L achieved 64% protection (Figure 6E). Effects of these formulations on the microfilariae were interesting (Figure 6F): in mice that became positive for microfilariae, vaccination with pMIP+pFlt3L reduced microfilaraemia by 90%; with pIL4+pMIP+pFlt3L, microfilariae counts were reduced 70%; however pIL4 caused a 1.5× increase in average microfilariae numbers in patent mice, which is consistent with our findings that IL-4 is associated with eosinophil-induced increase in worm fecundity in vaccinated animals [12], [42]. Despite considerable variability in worm recoveries we have always found (5 experimental repeats) that the comparison between pEmpty and the full vaccine has exceeded 65% reduction in microfilaraemia.

These data suggest that targeting immune modulatory proteins had protective effects against multiple parasite stages with substantial IL4-independent disruption of microfilariae production and gradual killing of adults, consistent with the finding that early disruption of Treg function during the onset of *L. sigmodontis* infection is sufficient to enhance microfilarial killing 60 days later [43].

### Immune determinants of protective immunity

**Figure 7:**
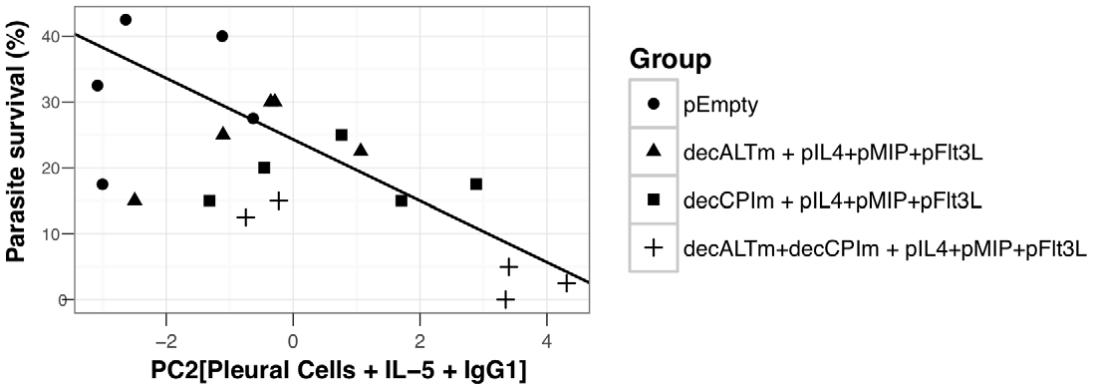
Protective immunity is mediated by IgG1 and pleural leukocyte recruitment. A principal component analysis was performed on 31 immunological measures from the mice in Figure 6C–D (see Table S1 and Figure S3 for PC selection) to identify immune responses associated with the killing of adult worms. The second component was most strongly associated with parasite-specific IgG1 and pleural leukocyte recruitment, and was the only component negatively correlated with parasite survival (r = −0.72, *P*<0.0001). pEmpty, non-coding plasmid control; decALTm, plasmid encoding the anti-DEC205 scFv-ALTm construct, decCPIm, plasmid encoding the anti-DEC205 scFv-CPIm construct, pIL4 pMIP and pFlt3L, plasmids expressing mouse IL-4, MIP-1α and Flt3L respectively. Points represent individual mice at D60 p.i. (N = 5 mice per group).

Many of the readouts assessed for this study did not reach statistical significance, perhaps due to high variability in both immune parameters and parasite numbers. We took statistical advantage of the substantial variation in both immune and parasitological read-outs to analyse the most prominent immune correlates of protection observed in Figure 6C. For example, pleural recruitment of eosinophils and parasite-specific IgG1 production were increased in the dual-vaccinated group (Figure S2B–C) although no significant effect on total IgE production was detected (Figure S2D). In total, thirty-one variables for each mouse (Table S1) were measured. To facilitate their analysis, we reduced them to principal components (PC) that summarise major patterns in the immune response. Three PC tested as significant and interpretable that captured 50% of the variation present in the full dataset (Table 1, Figure S3). Subsequent components were rejected for lack of explanatory power. The first component (PC1) included mainly lymph-node cell production of Th2 cytokines IL-5 and IL-13. PC2 included whole worm-specific IgG1, pleural IL-5, and pleural eosinophils, neutrophils, macrophages and less prominently, pleural lymphocytes. PC3 included mainly LsALT- and LsCPI2-specific IgG1, unstimulated IL-4 production by lymph node cells in vitro, anti-CD3 induced production of IFN-γ and pleural lymphocyte numbers (see Table S1 for individual rotation values). The explanatory power of the resulting components for parasite survival was assessed by GLM. Parasite numbers were affected by only the second principal component PC2, revealing a strong negative correlation between PC2 and parasite survival (Figure 7). An analysis of variance confirmed that the decALTm+decCPIm vaccine formulation drove most of the variation in PC2 (Figure 7).

**Table 1:** Importance of principal components of immunological responses.

	PC1	PC2	PC3
**Standard deviation**	2.657	2.197	1.902
**Proportion of Variance**	0.228	0.156	0.117
**Cumulative Proportion**	0.228	0.384	0.500

The first four components obtained from a PCA analysis of 31 immunological factors measured in vaccinated and challenged mice captured 56.5% of the total variation present in the full dataset. For the detailed contributions of the immunological factors to each principal component, please see Table S1.

Because IgE was weakly represented in those PCs while IgG1 was strongly represented in PC2, we wanted to determine the relative contribution of IgE, IgG1 and IgG2a concentrations to parasite killing. This confirmed that only IgG1 had a significant effect on parasite numbers (*P* = 0.015). Likewise, the analysis of respective roles for pleural cell types in protection revealed that parasite killing was significantly affected by pleural lymphocytes and neutrophils (*P* = 0.002 and *P* = 0.03 respectively), but only marginally by eosinophil numbers (*P* = 0.07) despite a significant negative correlation between eosinophil and parasite numbers (r = −0.5, *P* = 0.02). Taken together, these data are strongly suggestive of IgG1 and pleural leukocytes as being the main effectors in the decALTm+decCPIm vaccine-induced parasite killing.

## Discussion

Like many parasitic helminths, filarial nematodes establish long-lasting infections that are facilitated by immunomodulatory products secreted by the parasite [7]. Host-driven immune down-regulation can contribute to parasite survival [13], while in other patients, anti-filarial responses to adult and juvenile parasites are associated with immunopathology. Indeed, since immunity to helminths is mainly mediated by Th2-type responses, the risk of a vaccine generating excessive eosinophilia, IgE-mediated mast cell degranulation and related pathologies must be considered carefully [44]. Finally, we have previously demonstrated that filarial nematodes are able to adapt their developmental schedule to the hosts's eosinophilic response, thereby shortening their time to transmission [12]. We thus inferred that a successful vaccine against filarial infections would need to: (a) evoke a Th2 response, possibly through a path different from that driven by natural challenge; (b) target the parasite molecules that suppress protective immunity; (c), avoid inducing immune hyper-responsiveness. This suggested that the best vaccine candidates would be found among excreted/secreted molecules rather than structural components.

We selected parasite antigens based on their abundance in gene expression profiles [25], [26], potential to induce protective immunity [31], [45], and on their role in immune modulation as supported by *in vitro* studies [8], [9], and decided to take advantage of the flexibility and ease of production of DNA vaccination. Administering plasmids encoding the native sequences of LsALT and LsCPI failed to generate strong specific immune responses in mice, perhaps because of the immunomodulatory properties of these proteins when directly expressed in eukaryotic cells. This differs from other DNA vaccination studies in which ALT from the human parasite *B. malayi* induced a good response in rodents following challenge [17], [27], [28]. It may be that the immune modulatory properties of ALT are host-specific and are more readily manifested in the permissive host-parasite combination used in our study. Indeed our finding that when LsALT and LsCPI were genetically modified to remove immune modulatory residues, specific antibody responses increased, provides *in vivo* support to the *in vitro* evidence that these domains are immunosuppressive [8], [9]. It further indicates that the immunosuppressive function of these proteins can be successfully removed *in vivo*, thereby allowing vaccines that contain them to generate significant protection against multiple stages of the parasite.

Simultaneously with the plasmids expressing parasite antigens, we administered plasmids expressing host cytokines IL-4 to enhance Th2 responses required for the elimination of filarial infections [5], as well as MIP-1α and Flt3L to enhance activation and recruitment of dendritic cells [19], [46], [47]. In addition, a sequence encoding a single-chain Fv antibody directed against the dendritic cell surface marker DEC-205 [19] was added to the parasite sequence in order to maximise the uptake of antigen by the DCs recruited by MIP-1α, Flt3L and the injections. We hypothesise that it is the combination of these steps that lead to successful protection against infection. Further, our results demonstrate the feasibility of combining different adjuvant systems in a single vaccine to increase its efficacy. However, our parasitological data display substantial variability, especially in the rate of adult worm killing. This variability is inherent to the study system, and is consistent with that observed in natural filarial infections [41], [48]–[50].

A recurring problem with DNA immunisation has been the lack of protective immunity despite the ability to generate specific immune responses [1]. The gold standard of vaccination against filarial nematodes is immunisation with irradiated larvae, and requires the presence of functional eosinophils and antibody [51], [52]. A multivariate analysis of the immune factors that lead to protection in our present study revealed a negative correlation between whole parasite-specific IgG1 (measured in the blood) and the numbers of leukocytes at the site of infection, implying that immune effectors were correctly potentiated during the immunisation phase. Intriguingly, serum concentrations of IgE did not correlate with parasite numbers, despite being enhanced by the most protective vaccine regimens, and mediating protection in another rodent model of vaccine-mediated L3 killing [52]. A further consequence of this finding is that IgG1 and antigen-specific T cell responses may be sufficient to assess immunisation efficacy and the generation of protective immunity, but that IgE may not be an accurate marker for protection in mice, which may be due to the lack of Fc epsilon receptors on murine eosinophils [53]. The more prominent association between neutrophilia and protection is in accordance with other studies showing their role in secondary immunity to *Strongyloides stercoralis* in mice [54]. Further, our data suggest that in addition to the importance of targeting the parasite directly, the choice of adjuvants is crucial to generating immune responses that are protective. The coinjection of IL-4, MIP-1α and Flt3L lead to substantial reductions in parasite numbers, which was maintained or even improved when only MIP-1α and Flt3L were administered. Further refinements of this vaccine formulation are underway, but it currently appears that both DEC205 and MIP-1α+Flt3L are needed but the IL-4 may be dispensable.

In conclusion, strategic use of DNA vaccine technology has allowed us to test a large number of parameters and combinations of immune modulators that would not have been logistically possible if we needed to produce all the individual recombinant proteins in active form. This study provides a proof-of-principle that targeting parasite products that suppress the immune responses of their hosts while enhancing antigen presentation can lead to significant protection. Anti-evasion immunisation is garnering an increasing amount of attention in a wide range of pathogenic systems [55]–[58]. Our study shows that it is crucial and feasible to ablate the immunomodulatory function of such candidates for them to generate protective immune responses and we expect that this strategy may be applied to a wide range of diseases. Whether or not successful immunisation against filarial and other pathogens includes DNA vaccination, the work described here provides an approach to define the antigens and modulators most likely to generate very high levels of protection against all stages of infection, along with the ability to define the best correlates of protection.

## Supporting Information

Figure S1 **mRNA expression from plasmids injected to the muscle.** To verify that plasmids were successfully transcribed in the muscle, RT-PCR and cDNA amplification with specific primers were performed on mice 28 days after the second immunisation. Samples from two experiments and 3 mice for each group are shown. (A and B) ALTm and OVA mRNA expression respectively. Lanes 1–3: mice immunised with decALTm; lanes 4–6: mice immunised with isoOVA; lanes 7–9: Naive mice (C and D) CPI or CPIm, and OVA mRNA expression, respectively. Lanes 1–3: mice immunised with decCPI; lanes 4–6: mice immunised with decCPIm; lanes 7–9: mice immunised with isoOVA; lanes 10–12: Naive mice. See Table S3 for expression in other organs.
(TIFF)

Figure S2 **Increased antibody production against** ***L. sigmodontis*** **soluble antigen and CPI when DCs are targeted.** (A) LsCPI-specific IgG2a concentrations in the serum of mice were immunised with plasmids encoding either the native protein (CPI) or a plasmid expressing its mutated form (CPIm), in combination with pIL4+pFlt3L+pMIP or alone. All mice other than naïve were challenged with live infective *L. sigmodontis* larvae (GLM, *P*_decCPIm, [CPI, decCPI, CPIm, isoOVA]_ = 0.002, 0.2, 0.6, 0,001). (B) Whole parasite-specific IgG1 titres, (C), numbers of eosinophils in the pleural cavity, and (D) total IgE concentrations in mice immunised with decCPIm and decCPIm in addition to the adjuvant cocktail pIL4+pFlt3L+pMIP 60 days post-challenge (see Figure 7). pEmpty, non-coding plasmid control; decALTm, plasmid encoding the anti-DEC205 scFv-ALTm construct, decCPIm, plasmid encoding the anti-DEC205 scFv-CPIm construct. Points represent individual mice (N = 5 per group).
(TIFF)

Figure S3 **Selection of interpretable principal components.** The broken-stick criterion was used to select the most significant components. PC1 through 3 captured 50% of the variation present in the full dataset, and were then used as explanatory factors in a GLM with parasite numbers as the response variable. Detailed methods are presented in the data analysis section of Materials and Methods.
(TIFF)

Table S1 **Rotation of significant principal components.** Rotations of the first four principal components obtained from a PCA analysis of 31 immunological factors measured in vaccinated and challenged mice. The major contributing factors (underlined) were used to identify the immunological function most closely conveyed by each PC.
(DOC)

Table S2 **Primers used for cloning** ***Litomosoides sigmodontis*** **and mouse genes.**
(DOC)

Table S3 **Tissue distribution of ALT, ALTm, OVA, CPI and CPIm vaccines in mice by RT-PCR.** To assess where plasmids that were injected intramuscularly were expressed, RT-PCR and cDNA amplification with specific primers were performed on muscle, spleen, lungs and liver of mice 28 days after the second immunisation. Average abundance on a scale from 1 to 3 was estimated on a 1% agarose gel after PCR from the same mass of first strand cDNA, since quantitative PCR failed to detect some of the samples. Samples from two experiments and 3 mice for each group are shown.
(DOC)
