# Supplementary figures and images for: Deletion of Parasite Immune Modulatory Sequences Combined with Immune Activating Signals Enhances Vaccine Mediated Protection against Filarial Nematodes
Source: PLoS Negl Trop Dis. 2012 Dec 27;6(12):e1968. doi: 10.1371/journal.pntd.0001968 (PMC3531514; doi:10.1371/journal.pntd.0001968)

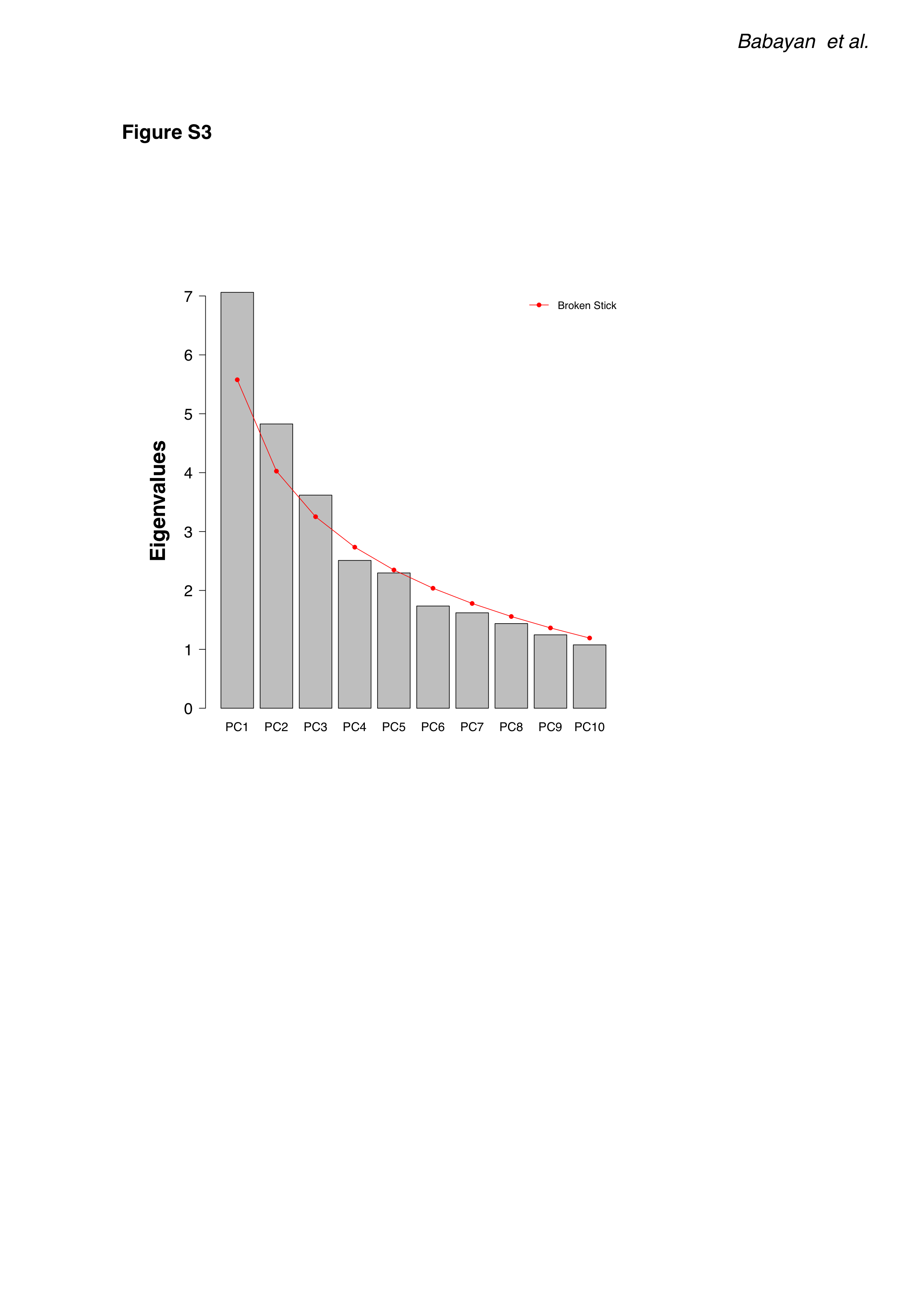

Supplement: Figure S3 — Selection of interpretable principal components. The broken-stick criterion was used to select the most significant components. PC1 through 3 captured 50% of the variation present in the full dataset, and were then used as explanatory factors in a GLM with parasite numbers as the response variable. Detailed methods are presented in the data analysis section of Materials and Methods. (TIFF) [file pntd.0001968.s003.tiff]
