# Supplementary material for: Deletion of Parasite Immune Modulatory Sequences Combined with Immune Activating Signals Enhances Vaccine Mediated Protection against Filarial Nematodes
Source: PLoS Negl Trop Dis. 2012 Dec 27;6(12):e1968. doi: 10.1371/journal.pntd.0001968 (PMC3531514; doi:10.1371/journal.pntd.0001968)
Supplement: Table S1 — Rotation of significant principal components. Rotations of the first four principal components obtained from a PCA analysis of 31 immunological factors measured in vaccinated and challenged mice. The major contributing factors (underlined) were used to identify the immunological function most closely conveyed by each PC. (DOC) [file pntd.0001968.s004.doc]

## Table S1: Rotation of significant principal component

|  | **PC1** | **PC2** | **PC3** |
| --- | --- | --- | --- |
| **Total IgE** | 0.0585 | -0.0187 | 0.0103 |
| **LsAg-specific IgG1** | -0.0241 | 0.2358 | -0.0165 |
| **LsALT-specific IgG1** | 0.1537 | -0.1685 | 0.3402 |
| **LsCPI-specific IgG1** | 0.1477 | -0.1841 | 0.3574 |
| **LsAg IgG2a** | 0.0371 | 0.0555 | -0.0319 |
| **LsALT-specific IgG2a** | 0.1872 | -0.0231 | -0.0643 |
| **LsCPI-specific IgG2a** | 0.1775 | -0.0538 | -0.2165 |
| **Pleural IL4** | -0.0126 | -0.1482 | -0.2109 |
| **Pleural IL5** | 0.0301 | 0.2220 | -0.2065 |
| **Pleural IL13** | 0.0307 | 0.1797 | -0.2815 |
| **Pleural IFNg** | -0.0392 | 0.0012 | -0.1745 |
| **Media-LN IL4** | 0.2033 | -0.0804 | 0.3057 |
| **LsAg-LN IL4** | 0.1936 | 0.0919 | -0.0417 |
| **aCD3-LN IL4** | 0.0659 | 0.1362 | 0.1495 |
| **Media-LN IL5** | 0.3342 | 0.0325 | 0.0726 |
| **LsAg-LN IL5** | 0.2992 | 0.1363 | -0.1185 |
| **aCD3-LN IL5** | 0.3134 | 0.0636 | -0.0931 |
| **Media-LN IL13** | 0.2144 | 0.0679 | 0.0304 |
| **LsAg-LN IL13** | 0.3120 | 0.1668 | -0.1365 |
| **aCD3-LN IL13** | 0.3068 | 0.0436 | -0.0756 |
| **Media-LN IFNg** | 0.2541 | 0.0707 | 0.1347 |
| **LsAg-LN IFNg** | 0.0675 | 0.0944 | -0.2149 |
| **aCD3-LN IFNg** | 0.1278 | -0.1507 | 0.3035 |
| **Pleural Eosinophils** | -0.0303 | 0.3795 | 0.1455 |
| **Pleural Neutrophils** | -0.1151 | 0.3569 | 0.1584 |
| **Pleural Macrophages** | -0.0384 | 0.3816 | 0.1557 |
| **Pleural Lymphocytes** | -0.1768 | 0.2057 | 0.2375 |
| **Total pleural cells** | -0.0910 | 0.3922 | 0.1994 |
| **Proliferation (Media)** | 0.2262 | 0.0024 | 0.1029 |
| **Proliferation (LsAg)** | 0.2252 | 0.1642 | -0.0443 |
| **Proliferation (aCD3)** | 0.1286 | -0.0591 | 0.0284 |
