# Supplementary material for: Deletion of Parasite Immune Modulatory Sequences Combined with Immune Activating Signals Enhances Vaccine Mediated Protection against Filarial Nematodes
Source: PLoS Negl Trop Dis. 2012 Dec 27;6(12):e1968. doi: 10.1371/journal.pntd.0001968 (PMC3531514; doi:10.1371/journal.pntd.0001968)
Supplement: Table S2 — Primers used for cloning Litomosoides sigmodontis and mouse genes. (DOC) [file pntd.0001968.s005.doc]

## Table S2: Primers used for cloning **Litomosoides sigmodontis** and mouse genes

| **Transcript** | **Primer sequence** | **Product size (bp)** |
| --- | --- | --- |
| **ALT** | F: 5'-CACCATGATGTCGGTGAAGGGTGTATTATT-3'  R: 5'-TTACGAAAGTAACTTTGTTTCCAAG-3' | 444 |
| **ALTm** | F:5'-CACCATGAACAAAGTTTTGATAATCTTTGGC-3'  R:5'-TTAATCGTATGAGCATT-3' | 306 |
| **LsCPI2[asn66] -> CPIm[lys66]** | F: 5'-GATAATCAACAGTCAAAAGATGCGTATCACCTTATGCC-3'  R: 5'-GGCATAAGGTGATACGCATCTTTTGACTGTTGATTATC-3' | NA |
| **CPI / CPIm** | F: 5'-ATGATGTCGGTGAAGGGTGTATTATT-3'  R: 5'-TTACGAAAGTAACTTTGTTTCCAAG-3' | 447 |
| **IL-4** | F: 5’-CACCATGGGTCTCAACCCCCAGCTAG-3’  R: 5’-CTACGAGTAATCCATTTGCATG-3’ | 423 |
| **Flt3-L** | F: 5’-CACCATGACAGTGCTGGCGCCAGCCT-3’  R: 5’-CTAGGGATGGGAGGGGAGGGGCAC-3’ | 699 |
| **MIP-1α** | F: 5’-CACCATGAAGGTCTCCACCACTGCCC-3’  R: 5’-TCAGGCATTCAGTTCCAGGTCA-3’ | 279 |
