# Supplementary material for: Deletion of Parasite Immune Modulatory Sequences Combined with Immune Activating Signals Enhances Vaccine Mediated Protection against Filarial Nematodes
Source: PLoS Negl Trop Dis. 2012 Dec 27;6(12):e1968. doi: 10.1371/journal.pntd.0001968 (PMC3531514; doi:10.1371/journal.pntd.0001968)
Supplement: Table S3 — Tissue distribution of ALT, ALTm, OVA, CPI and CPIm vaccines in mice by RT-PCR. To assess where plasmids that were injected intramuscularly were expressed, RT-PCR and cDNA amplification with specific primers were performed on muscle, spleen, lungs and liver of mice 28 days after the second immunisation. Average abundance on a scale from 1 to 3 was estimated on a 1% agarose gel after PCR from the same mass of first strand cDNA, since quantitative PCR failed to detect some of the samples. Samples from two experiments and 3 mice for each group are shown. (DOC) [file pntd.0001968.s006.doc]

**Table S3:Tissue distribution of ALT, ALTm, OVA, CPI and CPIm vaccines in mice by RT-PCR.**

| **Plasmid** | **Muscle** | **Spleen** | **Lungs** | **Liver** |
| --- | --- | --- | --- | --- |
| **ALT** | +++ | ++ | ++ | + |
| **ALTm** | +++ | ++ | ++ | + |
| **decALTm** | +++ | ++ | ++ | + |
| **isoALTm** | +++ | ++ | ++ | + |
| **isoOVA** | + | ++ | + | - |
| **CPI** | +++ | ++ | + | +++ |
| **decCPI** | +++ | ++ | + | +++ |
| **CPIm** | +++ | ++ | + | +++ |
| **decCPIm** | +++ | ++ | + | +++ |
| **Naïve** | - | - | - | - |
